# Supplementary material for: Mapping the response of primary human chondrocytes to an osteoarthritis-relevant stimulus by single-cell RNA sequencing
Source: bioRxiv. 2026 Sep 29:2026.09.24.754148. Preprint. [Version 1] doi: 10.64898/2026.09.24.754148 (PMC13648385; doi:10.64898/2026.09.24.754148)
Supplement: Supplementary file 1 [file NIHPP2026.09.24.754148v1-supplement-1.pdf]

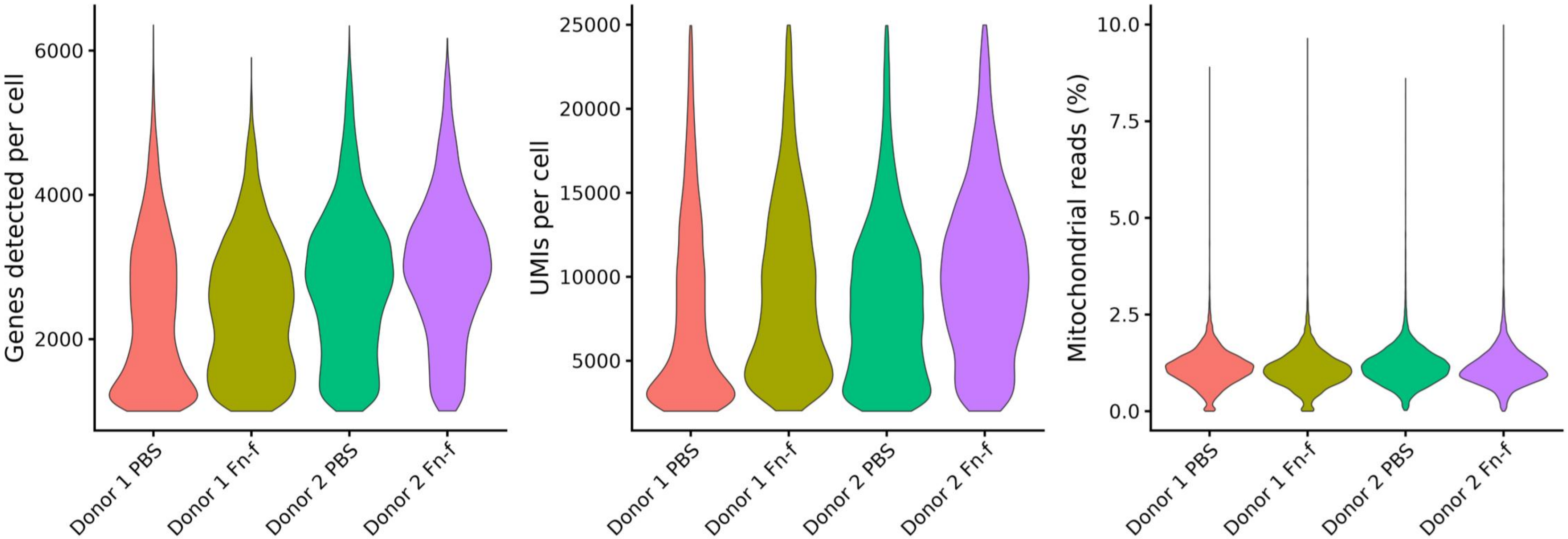

Donor	Treatment	Total reads	Reads mapped (%)	Sequencing saturation (%)	Number cells post QC	Median genes/cell	Median UMIs/cell	Median mitochondrial reads (%)
1	PBS	500,959,424	86.87	64	11,297	2,239	6,350	1.11
1	Fn-f	283,589,969	87.86	64	6,171	2,392	8,387	1.05
2	PBS	592,654,020	90.19	70	10,548	2,787	8,102	1.13
2	Fn-f	491,365,661	91.95	75	4,729	3,009	10,035	1.06

bioRxiv preprint doi: https://doi.org/10.64898/2026.09.24.754148; this version posted September 29, 2026. The copyright holder for this preprint (which was not certified by peer review) is the author/funder, who has granted bioRxiv a license to display the preprint in perpetuity. It is made available under a CC-BY 4.0 International license.

Supplementary Figure S1. Sequencing and quality-control metrics for single-cell RNA-seq samples.

Violin plots show the distributions of genes detected per cell, UMI counts per cell, and mitochondrial RNA percentage among cells retained following quality-control filtering. The table summarizes sequencing and cell-level quality-control metrics for each donor and treatment condition. Total reads, mapped reads, and sequencing saturation were obtained from DRAGEN Single Cell RNA processing metrics; cell counts and cell-level metrics reflect cells retained following Seurat filtering.
